# Effect of bronchofiberscopic lavage with acetylcysteine instillation on refractory mycoplasma pneumoniae pneumonia in children: a retrospective clinical observation

**DOI:** 10.1186/s13052-025-01879-y

**Published:** 2025-02-28

**Authors:** Pei-long Li, Hong-min Fu, Kai Liu, Feng Li, Jia-wu Yang

**Affiliations:** 1 Kunming Children’s Hospital & Children’s Hospital Affiliated to Kunming Medical University, Kunming Medical University, No. 28, Shulin Street, Xishan District, Kunming, China; 2 Department of Pulmonary and Critical Care Medicine, Yunnan Key Laboratory of Children’s Major Disease Research, Yunnan Medical Center for Pediatric Diseases, Kunming Children’s Hospital, Kunming Medical University, No. 28, Shulin Street, Xishan District, Kunming, China

**Keywords:** Refractory mycoplasma pneumonia, Bronchofiberscopic lavage, Acetylcysteine, Children, Immune function

## Abstract

**Background:**

Bronchofiberscopic lavage is a therapeutic treatment for mycoplasma infection. However, there are limited reports on the use of bronchofiberscopic lavage with acetylcysteine instillation. The aim of our study was to investigate the clinical effect of bronchofiberscopic lavage with acetylcysteine instillation on refractory mycoplasma pneumoniae pneumonia in children.

**Methods:**

This was a retrospective study. Clinical and laboratory data were collected from 280 children hospitalized with MPP in the Department of Pulmonary and Critical Care Medicine at Kunming Children′s Hospital from January 2021 to January 2023, and these were divided into two groups: the study group and the control group. Children in the control group were treated with anti-infective, antitussive, and antipyretic routine symptomatic treatment and nebulization of acetylcysteine suspension, while children in the study group were treated with bronchofiberscopic lavage with acetylcysteine instillation along with standard treatment as in the control group. The clinical characteristics, adverse reactions, pulmonary imaging changes, and laboratory examination indicators were compared between the two groups.

**Results:**

The treatment effective rate and the absorption area of lung lesions in the study group were significantly higher than those in the control group (*P* < 0.05). Before treatment, there were no statistically significant differences in WBC, IgG, IgE, CRP, and IL-6 levels between the two groups (*P* > 0.05). After 7 days of treatment, IgG levels in both groups were higher than those before treatment, while WBC, IgE, CRP, and IL-6 levels were lower than those before treatment (*P* < 0.05).

**Conclusions:**

The bronchofiberscopic lavage with acetylcysteine instillation constitutes a safe and efficacious therapeutic regimen, which might alleviate the clinical symptoms experienced and reduce the levels of inflammatory factors for children with RMPP.

## Introduction

During the pandemic of the novel coronavirus (COVID-19), the heightened global preventive measures effectively suppressed the transmission of other pathogens, including Mycoplasma pneumoniae (MP). Consequently, the infection rate of MP dropped to exceptionally low levels [1]. However, in the post-pandemic era, many countries and regions have observed a notable resurgence in MP infection rates, accompanied by a surge in hospitalizations that have even surpassed pre-pandemic levels [2]. Additionally, the increasing resistance of MP towards macrolides has contributed to in the heightened prevalence of Mycoplasma Pneumoniae Pneumonia (MPP) in China [3].

MPP, as an interstitial pneumonia caused by mycoplasma pneumoniae infection, is one of the most common respiratory infections encountered in clinical practice [4]. However, with the increase of resistance to macrolide antibiotics, conventional macrolide antibiotics have limited efficacy in the treatment of MPP [5]. As the condition deteriorates, many children gradually develop Refractory Mycoplasma Pneumoniae Pneumonia (RMPP) with atelectasis and pulmonary consolidation, causing substantial economic and societal burdens. In recent years, with the advancement of bronchoscopy technology, the therapeutic value of alveolar lavage on infected sites has been widely recognized. Bronchoscopy allows for the rinsing or injection of medications into the segmental or subsegmental bronchi [6]. Stimulating the local airway mucosa enhances the patient’s sputum excretion and suction of inflammatory secretions. Acetylcysteine suspension is thought to have a reparative effect on inflammatory airway injury tissues, with minimal systemic effects and high safety. In China, acetylcysteine is one of the most regularly used medications for the lavage solution during bronchoscopic alveolar lavage, with fewer reports from other countries.

In this study, the clinical efficacy of bronchofiberscopic lavage with acetylcysteine instillation in the treatment of RMPP was investigated, and adverse reactions were observed.

## Materials and methods

### Study design and subjects

Between January 2021 and January 2023, the study was conducted to review a total of 280 pediatric patients with RMPP admitted to the Respiratory and Critical Care Medicine Department of Kunming Children’s Hospital. RMPP was defined as: (1) sputum culture, serum etiology and imaging of the lungs suggested mycoplasma pneumoniae pneumonia in children; (2) persistent fever for 7 days or more; and (3) worsening cough and infiltrates on chest radiographs despite administration of appropriate macrolide antibiotics. 172 patients were excluded from the bronchoscopic lavage treatment due to disagreement from their guardians and comprised the control group. The remaining 108 patients, whose parents provided consent and signed the surgical consent form for bronchoscopic lavage treatment with acetylcysteine, comprised the study group.

Inclusion criteria: (1) children who meet the diagnostic criteria for RMPP by imaging examination and laboratory-related instrumental examinations [7]; (2) children aged 1–14 years.

Exclusion criteria: (1) immunodeficiency disorders, cardiovascular disease; (2) recurrent respiratory infection, bronchopulmonary dysplasia, and bronchiolitis obliterans; (3) patients who are long-term users of immusuppressive medications; (4) patients with infections caused by Mycobacterium tuberculosis, adenovirus, syncytial virus, rhinovirus, fungi, bacteria, and other pathogens.

Both groups of children received conventional treatment, including symptomatic measures such as expectoration assistance, oxygen inhalation, respiratory support, nutritional support, and postural drainage. Additionally, Erythromycin was administered intravenously at a dose of 10–15 mg/kg, twice daily for 3 days. Following this, nebulization of acetylcysteine suspension was administered twice daily for 14 days.

For children in the study group, the treatment regimen was based on that of the control group. The treatment time of the study group was the same as that of the control group. Before surgery, The children were fasted for four to six hours. Nasal secretions were cleared to maintain a patent respiratory tract. sedationwas induced with midazolam (0.1–0.3 mg/kg), and atropine was administered intravenously at a dose of 0.01 mg/kg. During the surgery, 1% lidocaine was used for surface anesthesia of the bronchial mucosa. An Olympus BF-XP260F bronchoscope, with an outer diameter of 2.8 mm, was selected for the procedure. The bronchoscope was inserted nasally, navigating through the throat, vocal cords, and into the airways. Sequential inspection was conducted of the glottis, trachea, bronchi, and the abnormal areas.The saline at 37℃ was infused with a perfusion volume of 5–10 ml for each segment of the bronchus. Following each lavage, negative pressure suction was applied. For lung segments with large shadows or consolidated lungs, normal saline lavage was followed by the solution of acetylcysteine (6 ml). Throughout the surgery, oxygen therapy, blood oxygen saturation monitoring, and electrocardiographic monitoring were continuously conducted.

### Outcome measures

Criteria for curative effect: the therapeutic impact was assessed based on symptoms, lung imaging, and other relevant factors. The following criteria [8] were used:

1. Cured: fever, cough, expectoration and other clinical symptoms completely disappeared, and lung lesions observed on imaging have been absorbed by 80%.
2. Effective: cough, expectoration, and shortness of breath have decreased, with lung lesions observed on imaging being absorbed by 50%.
3. Ineffective: cough, expectoration, and shortness of breath have not improved or have worsened, and lung imaging has shown a change of 30% or more.

Before and after the treatment, 3 ml of fasting peripheral venous blood were collected from each patient, The blood samples were anticoagulated in EDTA vacuum tubes. Centrifugation was performed at room temperature using a high-speed refrigerated centrifuge, with a rotational speed of 3000 revolutions per minute for 15 min. Following serum separation, the concentrations of White Blood Cell (WBC), C Reactive Protein (CRP), IgG, IgE and Interleukin-6 (IL-6) were measured. The incidence of adverse reactions during treatment was monitored in the study groups.

### Statistical analysis

Statistical analyses for the study were conducted using SPSS 25.0 software. For quantitative variables, the mean (M) and standard deviation (SD) were calculated to assess central tendency and dispersion, respectively. Nominal and ordinal variables were summarized using frequency counts and percentages (%). When comparing more than two groups, one-way ANOVA was employed. A p-value of less than 0.05 was considered statistically significant.

## Results

The treatment effective rate for children in the study group was 97.2%, significantly higher than the 85.5% observed in the control group (*P* < 0.05), indicating a statistically significant difference between the two groups (Table 1).

**Table 1:** Comparison of treatment effective rate [n(%)]

Cinical efficacy [*n* (%)]	Study group(*n* = 108)	Control group (*n* = 172)	Statistical results
**Cured**	78(72.2)	92(53.5)	*χ²=*10.189 *P* < 0.001
**Effective**	27(25.0)	55(31.9)	/
**Ineffective**	3(2.8)	25(14.5)	
**Effective rate**	105(97.2)	147(85.5)	*χ²=*9.762 *P* < 0.001

### Characteristics of all the cases

There was no significant difference between the two groups regarding age and gender (*P* > 0.05). The antipyretic time, cough time, time of hospital stay in the study group were shorter than those in the control group (*P* < 0.05), with statistically significant differences (Table 2).

**Table 2:** Comparison of characteristics

Characteristics	Study group(*n* = 108)	Control group(*n* = 172)	Statistical results
**Age[year]**	5.53 ± 0.98	5.54 ± 0.98	*F* = 0.106 *P =* 0.916
**Gender[n(%)]**			
**Male**	49(45.4)	81(47.1)	*χ²=*0.079 *P =* 0.778
**Female**	59(54.6)	91(52.9)	
**Antipyretic time[d**,** x ± s]**	7.53 ± 0.98	8.53± 0.78	*F*=−2.930 *P* < 0.001
**Cough time[d**,** x ± s]**	5.64 ± 1.17	7.64± 1.07	*F*=−5.174 *P* < 0.001
**Time of hospital stay[d**,** x ± s]**	8.32 ± 1.17	9.32± 1.47	*F*=−4.651 *P* < 0.001

### Incidence of adverse reactions

The bronchoscopic procedures were successfully completed for all 108 pediatric patients in the study group. Among these children, 76 cases(70.4%) had no complications during or after the procedure, while 32 cases(29.6%) experienced complications of adverse reactions.The primary complications observed were transient hypoxemia and sinus tachycardia.Additionally, there were 12 cases (11.1%) of transient fever, which normalized after physical cooling measures ororal of antipyretic drugs. 6 cases (5.5%) experienced mild paroxysmal cough, and 3 cases (2.8%) had hoarseness, both of those quickly resolved after nebulized inhalation of budesonide. 5 cases (4.6%) experienced transient hypoxemia, which returned to normal after discontinuing the procedure and increasing the oxygen flow rate. 3 cases (2.8%) experienced bleeding, including nasal and bronchial mucosal bleeding, with all instances being minor in volume. Only one case required adrenaline for hemostasis. 7 cases (6.5%) of vomiting were observed, we took the measures withdrawn the bronchoscope immediately. The relief was achieved within ten seconds (Table 3).

**Table 3:** Occurrence of adverse reactions[n(%)]

Adverse reactions[*n* (%)]	Cases	Percentage
**Transient hypoxemia**	5	4.6%
**Vomiting**	7	6.5%
**Mild paroxysmal cough**	6	5.5%
**Transient fever**	12	11.1%
**bleeding**	3	2.8%

### Pulmonary imaging changes

**Fig. 1:**
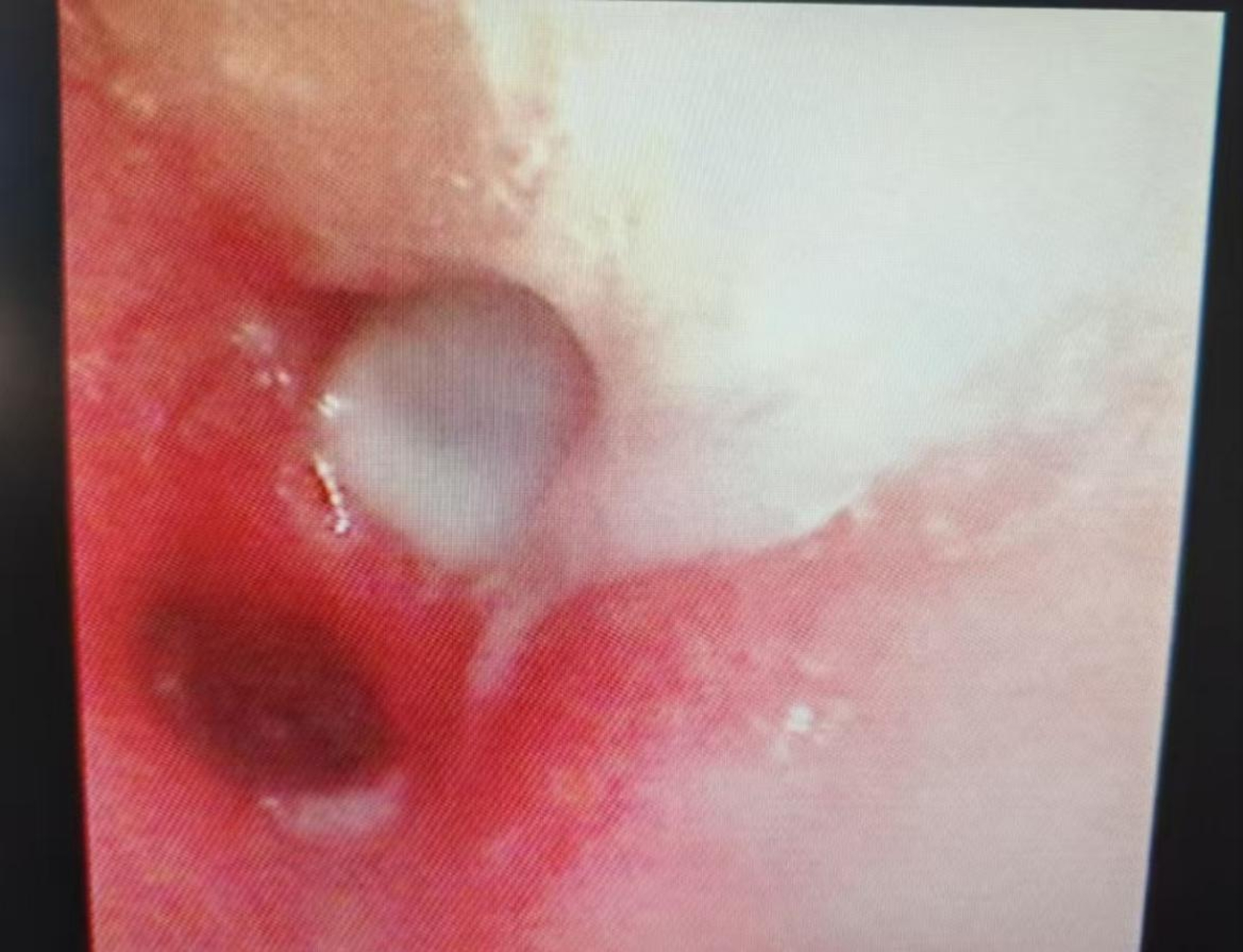
Sputum embolus

**Fig. 2:**
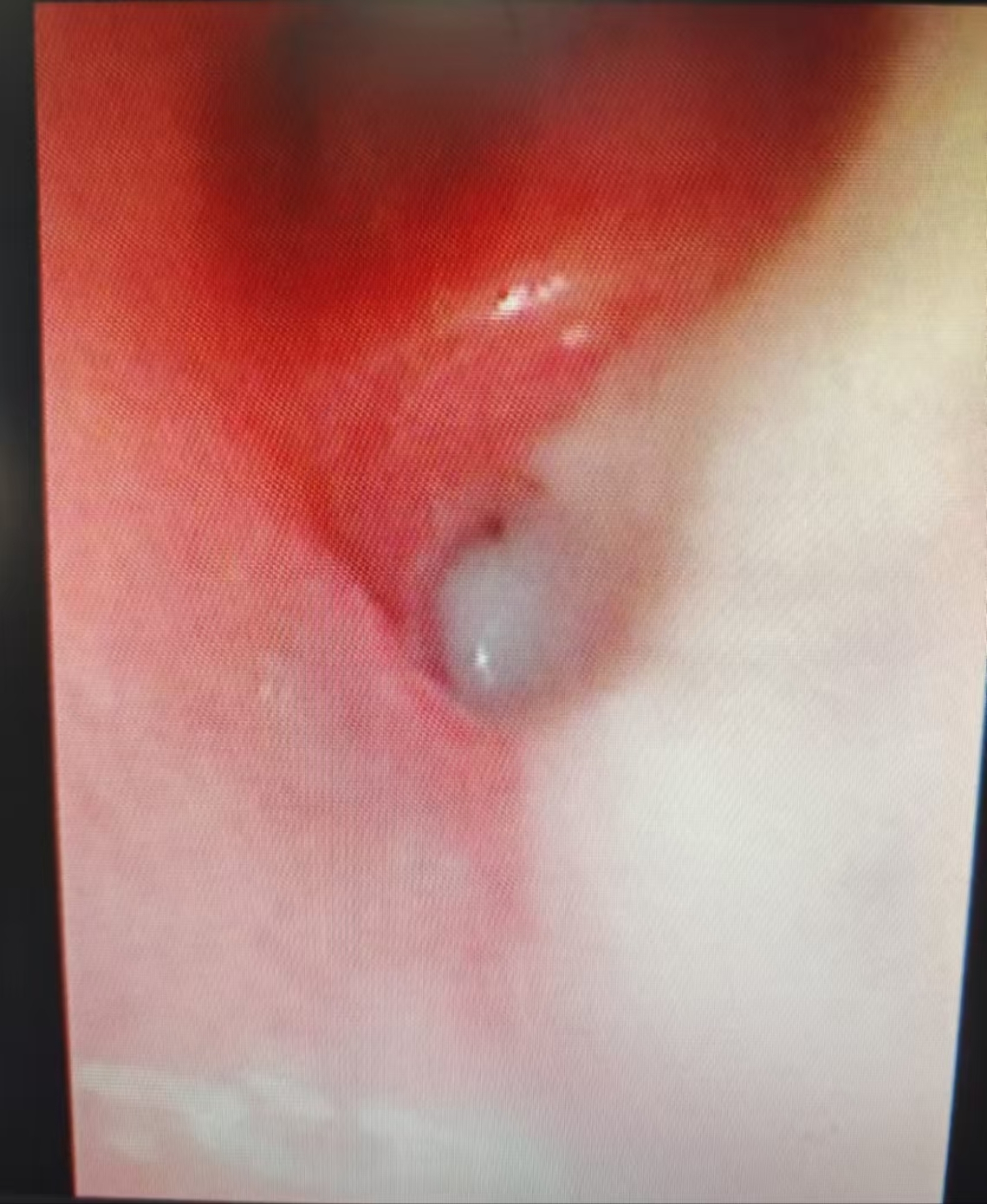
A mucous discharge originating from one branch of the bronchus

**Fig. 3:**
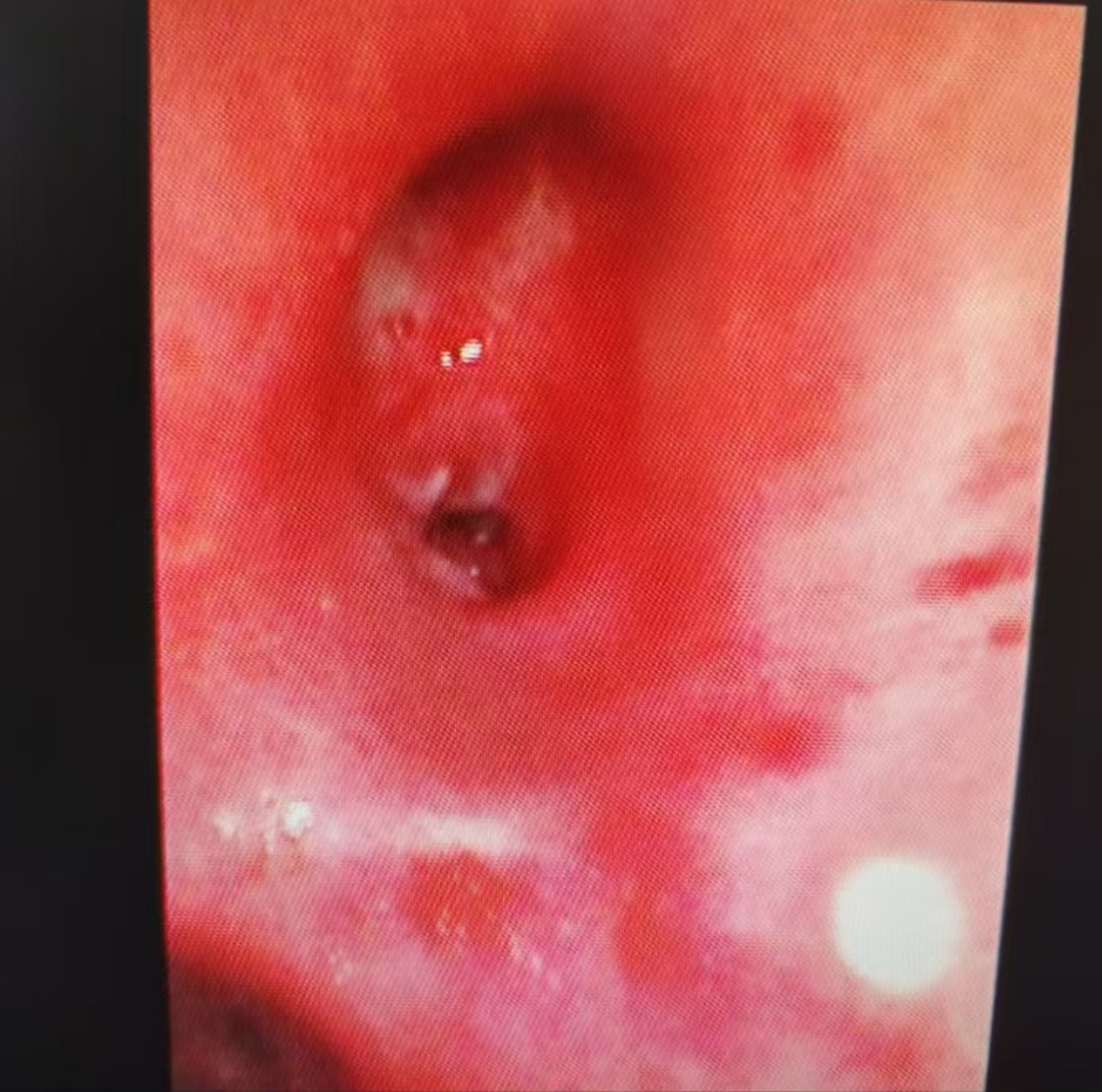
After a week of acetylcysteine instillation treatment, most of the sputum embolis were absorbed, but some edema persisted

**Fig. 4:**
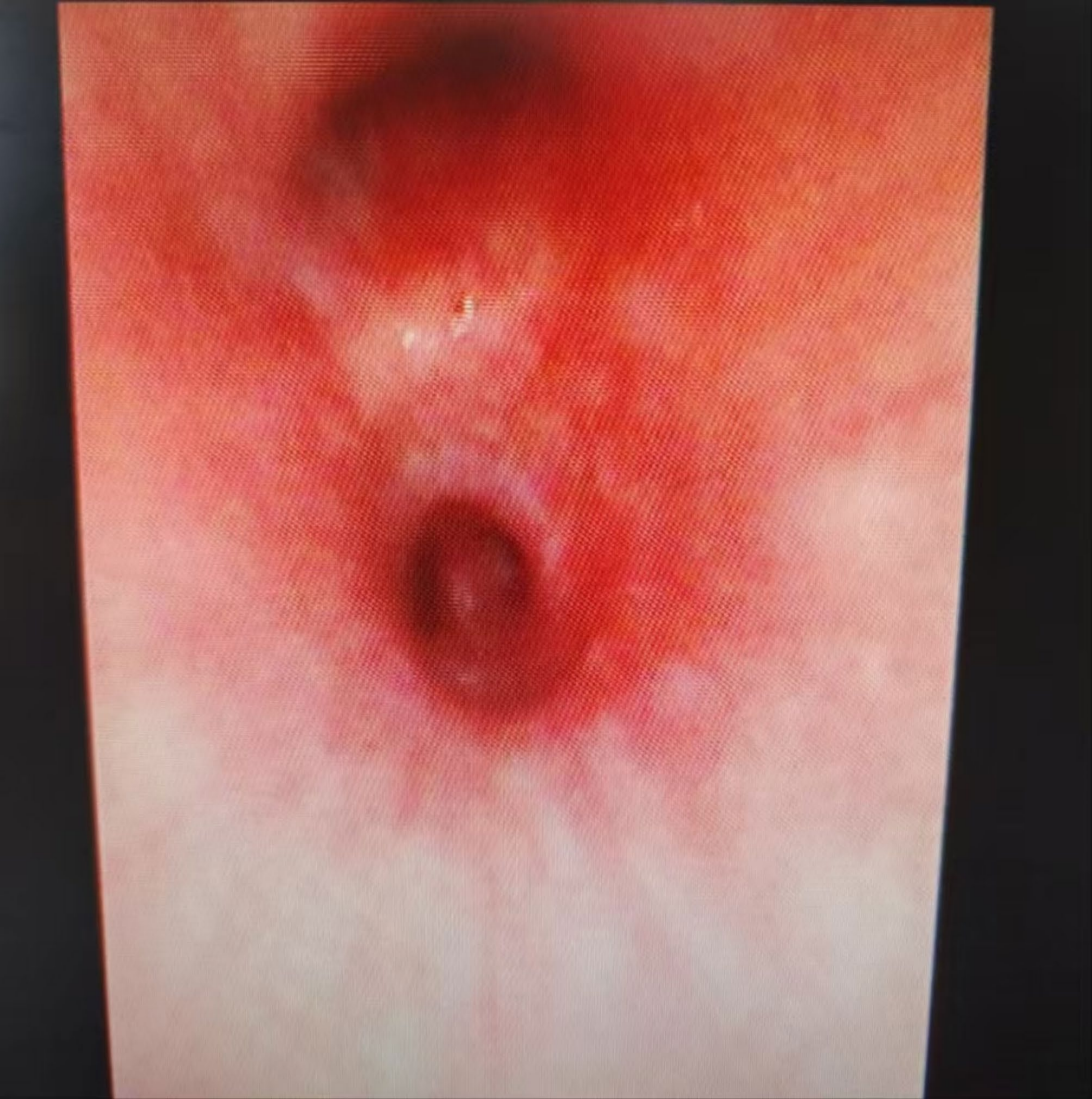
After two week of acetylcysteine instillation treatment, the sputum embolis were completely absorbed and the main airway was unobstructed

**Fig. 5:**
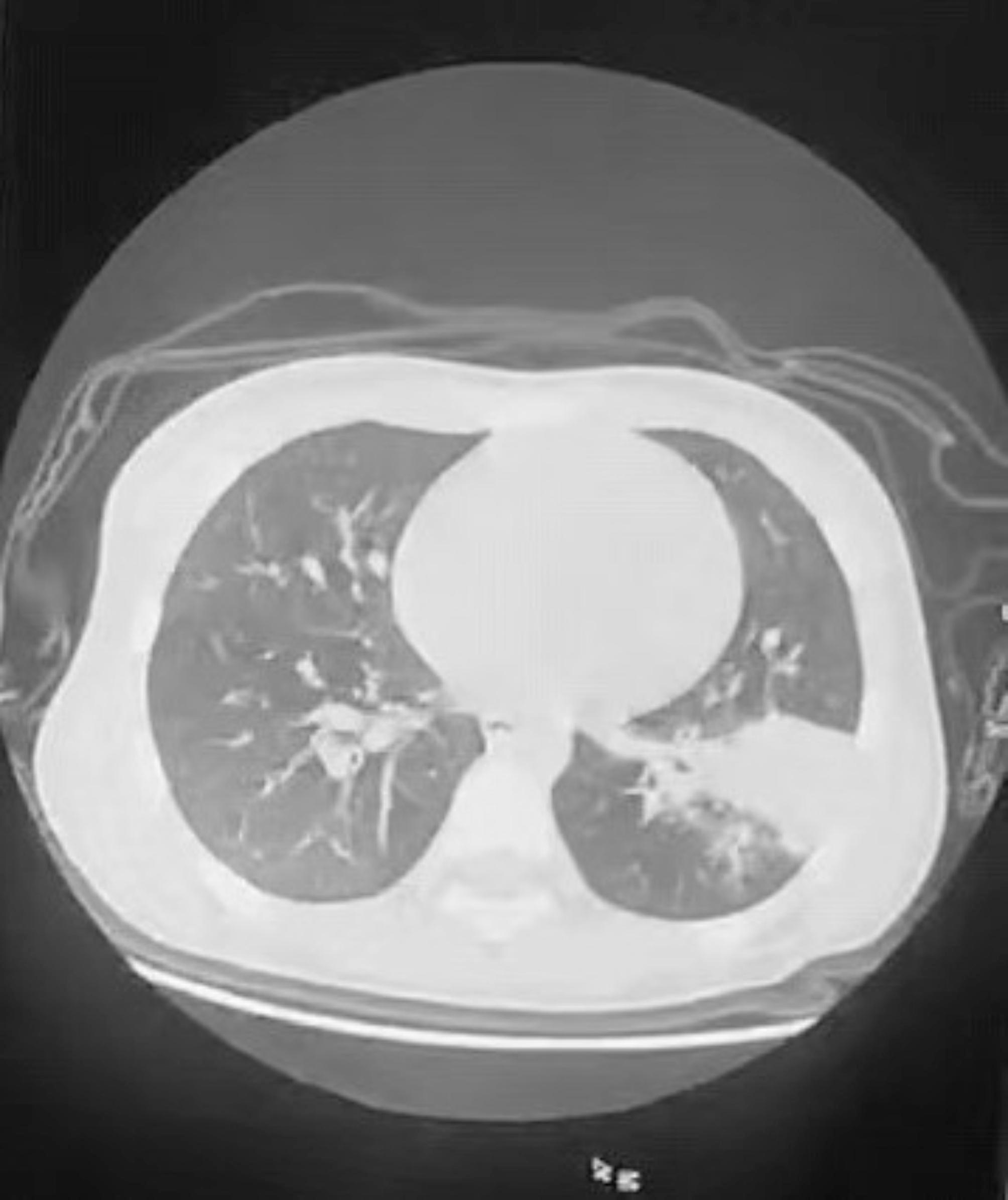
Bilateral pneumonia with prominent consolidation in the left lower lung

**Fig. 6:**
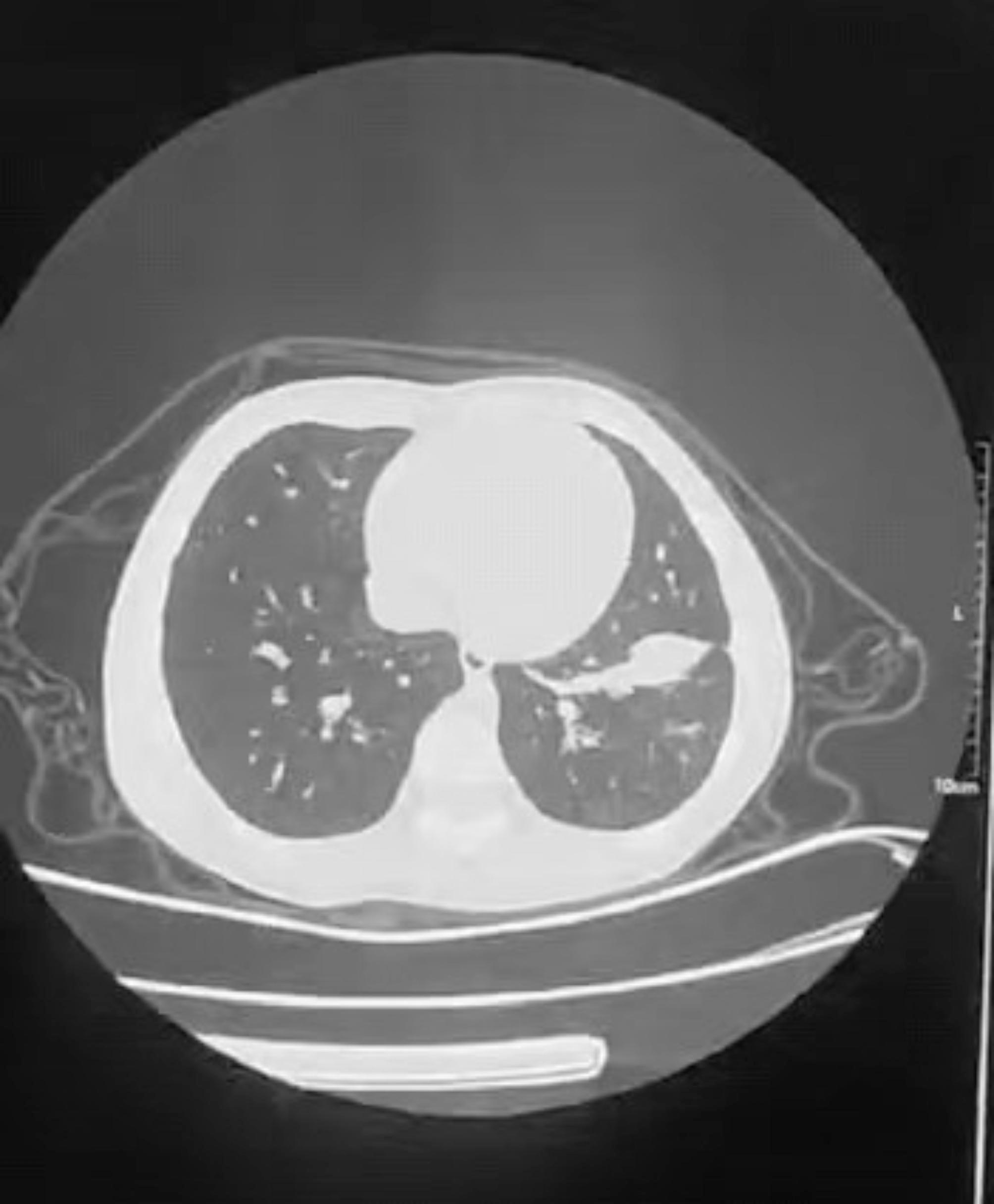
The pneumonia showed improvement after 2 weeks

After one week of therapy, the absorption area of lung lesions in the two groups differed significantly, the improvement rate in the study group was more significant than those in the control group (*P* < 0.05). Lung imaging revealed that acetylcysteine absorbed most inflammation two week after therapy. One patient with atelectasis in the study group was used pliers to extract the sputum embolus followed by acetylcysteine instillation. The bilateral pneumonia was entirely absorbed following imaging of the CT-scan after two week (Table 4) (Figs. 1, 2, 3, 4, 5 and 6).

**Table 4:** Comparison of the pulmonary imaging changes

Groups[*n*(%)]	One week of therapy			Two week after therapy			χ²	*P*
	Absorbed completely	Partial absorption	No change	Absorbed completely	Partial absorption	No change		
**Study group** **(n = 108)**	65	34	9	67	38	3	62.66*	< 0.001*
**Control group** **(n = 172)**	67	83	22	73	87	12	51.44*	< 0.001*

Annotate: *The changes in pulmonary imaging following one week and two week therapy were compared between the two groups

### Comparison of laboratory examination indicators

Before treatment, there were no statistically significant differences in WBC, IgG, IgE, CRP, and IL-6 levels between the two groups (*P* > 0.05). After 7 days of treatment, IgG levels in both groups were higher than those before treatment, while WBC, IgE, CRP, and IL-6 levels were lower than those before treatment (*P* < 0.05). Additionally, IgG levels in the study group were higher than those in the control group, and IgE levels in the study group were lower than those in the control group. Interestingly, the changes in laboratory indices following therapy were compared across groups, and the difference in IL-6, IgG were statistically significant (*P* < 0.05) (Table 5).

**Table 5:** Comparison of the laboratory examination indicators(x ± s)

Groups	CRP(mg/L)		WBC(10^9^/L)		IgE(g/L)		IgG(g/L)		IL-6(pg/mL)	
	Before treatment	After treatment	Before treatment	After treatment	Before treatment	After treatment	Before treatment	After treatment	Before treatment	After treatment
**Study group** **(n = 108)**	48.01 ± 11.67	8.37 ± 2.28	9.95 ± 3.3	7.73 ± 2.0	9.51 ± 1.84	2.41 ± 1.57	7.69 ± 0.88	12.67 ± 1.12*	39.57 ± 5.73	12.35 ± 2.03*
**Control group (n = 172)**	47.72 ± 12.08	11.19 ± 3.49	9.38 ± 2.88	6.61 ± 1.22	9.78 ± 1.91	3.19 ± 1.47	7.54 ± 0.91	9.59 ± 1.94	40.06 ± 5.86	19.78 ± 1.96
***t***	0.198	7.510	1.557	5.869	1.196	4.302	1.434	15.301	0.685	31.027
***P***	0.843	< 0.001	0.121	< 0.001	0.233	< 0.001	0.153	< 0.001	0.494	< 0.001

Annotate: *The changes in laboratory indices after treatment were compared across groups.and the difference in IL-6 and IgG was statistically significant (*P* < 0.05)

## Discussion

In the past few years, numerous countries and regions have successively reported outbreaks of MPP following the pandemic of the novel coronavirus (COVID-19) [9]. MPP, caused by the invasion of the respiratory tract by MP, leads to bronchial and lung damage. As the disease progresses and the drug resistance of pediatric patients increases, it can evolve into RMPP, further augmenting the complexity of treatment. Currently, in addition to conventional antibiotics and other off-label antibiotics, clinical practice primarily employs electronic bronchoscopy to remove excessive mucus from the bronchi to improve ventilation function in pediatric patients.

In this study, the total effective treatment rate in the study group was higher than that in the control group, The antipyretic time, cough time and time of hospital stay were shorter than those in the control group. These findings suggest that bronchofiberscopic lavage with acetylcysteine instillation with RMPP significantly improves clinical symptoms and reduces the time to the hospital for pediatric patients.The main component of acetylcysteine is N-acetyl-L-cysteine acid. Acetylcysteine possesses the ability to disrupt the disulfide bonds of glycoprotein within sputum, inhibiting the activity of alveolar proteinase. This action facilitates the breakdown of mucus and rapid dissolution of viscous secretions. Furthermore, acetylcysteine can inhibit the production of inflammatory factors, acting as an antioxidant, which could reduce the inflammatory response and minimize mucosal stimulation for infection prevention. In China, acetylcysteine is widely utilized to decrease sputum viscosity and stimulate expectoration in children. The effect of bronchofiberscopic lavage with acetylcysteine instillation has also received positive recognition in the treatment of RMPP.

In our study, the primary adverse reactions were transient fever, transient hypoxemia, mild paroxysmal cough, and vomiting during the treatment, as previously reported by Schramm et al. [10]. Following appropriate symptomatic treatment, these complications did not impact the overall course of therapy. The occurrence of transient hypoxemia during electronic bronchoscopic lavage indicates a low arterial oxygen saturation in pediatric patients [11], During the treatment, the prompt cessation of the lavage and administration of oxygen therapy are necessary. The presence of vomiting in the study group may be attributed to the spillage of acetylcysteine instillation as the electronic bronchoscope passes through the nasopharyngeal area. Additionally, azithromycin has the potential to cause gastrointestinal irritation [12]. The unique odor of the acetylcysteine may also induce a vomiting reflex in patients, leading to nausea and other related symptoms. Therefore, adhering to standardized operative procedures during the procedure can help prevent the solution from being sprayed into the throat. Based on our experience: Firstly, the purpose of performing bronchoscopy should be clearly defined. Before the examination, it is crucial to analyze and compare the patient’s condition with chest radiographs, CT scans, and anatomical diagrams. If bronchoalveolar lavage is indicated, the volume of lavage fluid should be kept within a reasonable range, typically ranging from 0.5 to 1.0 ml/kg. After lavage, the lavage fluid should be thoroughly aspirated and collected for further analysis. Then, it is essential to ensure that the pediatric patient has fasted for a sufficient period of time to prevent intraoperative aspiration and asphyxia. The choice of anesthesia method is crucial. Currently, there are two main anesthesia methods: local anesthesia and general anesthesia. In most hospitals of China, general anesthesia is the preferred method for bronchoscopy. However, Godfrey et al. [13] reported that bronchoscopic treatment being performed with intravenous midazolam for sedation. As for which anesthesia method is safer for bronchoscopy in children, further investigation is indeed necessary. As an operator, it is essential to have a clear understanding of the potential complications associated with bronchoscopy, to make timely and accurate judgments, and to handle them correctly. Additionally, it is crucial to develop effective emergency response plans in advance.

MPP predominantly manifests as interstitial lung disease on imaging, typically presenting as increased lung markings or scattered patchy shadows. In pediatric patients, pulmonary consolidation may also occur, with the right lung being the more frequently affected site. In this study, we observed one pediatric patient who exhibited pulmonary consolidation in the right lung. Notably, this patient showed significant improvement after treatment with acetylcysteine instillation. Studies have demonstrated that that MP can stimulate both humoral and cellular immunity, leading to disruptions in Ig antibodies and cytokines in children with RMPP [14], These disruptions exacerbate respiratory inflammatory damage. Serum immunoglobulins are commonly used as indicators for monitoring humoral immune function in such cases. As well, IL-6 is a multifaceted cytokine that exhibits a close relationship with the immune response, as well as playing crucial roles in the regulation and defense functions of the hematopoietic system. The levels of IL-6 positively correlate with the severity of infection and inflammation, and it plays a pivotal role in the pathogenic process of pneumonia infections [15]. In our study, we observed that WBC, CRP, and IL-6 levels decreased in both groups after treatment, these findings indicate that acetylcysteine instillation effectively inhibits the release of inflammatory mediators and cytokines. The variations in IL-6 and IgG were statistically significant after treatment. Therefore, the administration of acetylcysteine into the airways through electronic bronchoscopic lavage may have the potential to regulate humoral immune function, further elevate IgG levels and this treatment might help to reduce the risk of allergic reactions through decrease IgE levels. Moreover, this treatment also alleviates airway inflammation caused by abnormal immune responses and leading to a significant reduction in IL-6 level in children with RMPP, the specific mechanisms of action are not clear.

In clinical practice, clinical guidelines emphasize the importance of bronchoalveolar lavage as a treatment for pediatric patients diagnosed with lung consolidation accompanied by radiological evidence of sputum plugging, especially those with RMPP. Given the current trends of high MP infections worldwide, the use of off-label antibiotics (such as fluoroquinolones or tetracyclines) in pediatric patients poses significant concerns due to potential increased adverse effects [16], the emergence of antibiotic resistance and the inappropriate use of these drugs are remaining a significant concern [17]. Based on a comprehensive assessment of clinical presentation and the response to previous treatments of the patient, bronchofiberscopic lavage with acetylcysteine instillation may be a more appropriate treatment option for children when RMPP is diagnosed.

## Conclusions

Our research highlights the significance of bronchofiberscopic lavage with acetylcysteine instillation in reducing inflammatory reactions. Our study had some limitations, including a lack of flexibility and randomization, due to inadequate statistical power. This limitation could be resolved through prospective studys and increasing the number of participants. Therefore, future researches require more experimental and cohort studies to enhance the detection of a statistically significant effect.

## Data Availability

If reasonably requested, it can be obtained from the corresponding author.

## Abbreviations

MP: Mycoplasma Pneumoniae
MPP: Mycoplasma Pneumoniae Pneumonia
WBC: White Blood Cell
CRP: C Reactive Protein
IL-6: Interleukin-6
RMPP: Refractory Mycoplasma Pneumoniae Pneumonia
